# Machine learning application for development of a data-driven predictive model able to investigate quality of life scores in a rare disease

**DOI:** 10.1186/s13023-020-1305-0

**Published:** 2020-02-12

**Authors:** Ottavia Spiga, Vittoria Cicaloni, Cosimo Fiorini, Alfonso Trezza, Anna Visibelli, Lia Millucci, Giulia Bernardini, Andrea Bernini, Barbara Marzocchi, Daniela Braconi, Filippo Prischi, Annalisa Santucci

**Affiliations:** 1 Department of Information Engineering and Mathematics, University of Siena, Via Roma 56, 53100 Siena, Italy; 2 Toscana Life Sciences Foundation, Siena, Italy; 3 Energy way, Modena, Italy; 4 Department of Information Engineering and Mathematics, University of Siena, Siena, Italy; 5 UOC Patologia Clinica, Azienda Ospedaliera Senese, Siena, Italy; 6 School of Life Sciences, University of Essex, Colchester, CO4 3SQ UK

**Keywords:** Rare disease, Alkaptonuria, Machine learning, QoL scores, Precision medicine

## Abstract

**Background:**

Alkaptonuria (AKU) is an ultra-rare autosomal recessive disease caused by a mutation in the homogentisate 1,2-dioxygenase (HGD) gene. One of the main obstacles in studying AKU, and other ultra-rare diseases, is the lack of a standardized methodology to assess disease severity or response to treatment. Quality of Life scores (QoL) are a reliable way to monitor patients’ clinical condition and health status. QoL scores allow to monitor the evolution of diseases and assess the suitability of treatments by taking into account patients’ symptoms, general health status and care satisfaction. However, more comprehensive tools to study a complex and multi-systemic disease like AKU are needed. In this study, a Machine Learning (ML) approach was implemented with the aim to perform a prediction of QoL scores based on clinical data deposited in the ApreciseKUre, an AKU- dedicated database.

**Method:**

Data derived from 129 AKU patients have been firstly examined through a preliminary statistical analysis (Pearson correlation coefficient) to measure the linear correlation between 11 QoL scores. The variable importance in QoL scores prediction of 110 ApreciseKUre biomarkers has been then calculated using XGBoost, with K-nearest neighbours algorithm (k-NN) approach. Due to the limited number of data available, this model has been validated using surrogate data analysis.

**Results:**

We identified a direct correlation of 6 (age, Serum Amyloid A, Chitotriosidase, Advanced Oxidation Protein Products, S-thiolated proteins and Body Mass Index) out of 110 biomarkers with the QoL health status, in particular with the KOOS (Knee injury and Osteoarthritis Outcome Score) symptoms (Relative Absolute Error (RAE) 0.25). The error distribution of surrogate-model (RAE 0.38) was unequivocally higher than the true-model one (RAE of 0.25), confirming the consistency of our dataset. Our data showed that inflammation, oxidative stress, amyloidosis and lifestyle of patients correlates with the QoL scores for physical status, while no correlation between the biomarkers and patients’ mental health was present (RAE 1.1).

**Conclusions:**

This proof of principle study for rare diseases confirms the importance of database, allowing data management and analysis, which can be used to predict more effective treatments.

## Background

Alkaptonuria (AKU) was described by Garrod in 1908 [1] as the first disorder to conform with the principles of Mendelian recessive inheritance. The estimated incidence of AKU is 1 case in 250.000–1.000.000 births in most ethnic groups [2], with about 950 patients reported in 61 countries [3]. AKU patients carry homozygous or compound heterozygous mutations of the *HGD* gene leading to a deficiency of the enzyme homogentisate 1,2-dioxygenase (HGD), which is involved in the catabolic pathway of tyrosine [4, 5]. Such dysfunction causes accumulation of homogentisic acid (HGA). Most of HGA is excreted through the urine, resulting in the characteristic darkening-upon-standing, but smaller HGA amounts can also accumulate in connective tissues, where HGA polymerizes forming a dark brown melanin-like pigment (ochronotic pigment). Ochronosis affects skin, sclera and ears (presenting with blue-black discolouration), spine and joints (causing a dramatic degeneration and chronic inflammation), heart valves (leading to stenosis), and kidneys (where stones may develop) [2]. Ochronosis is also the main cause of arthropathy early onset, severely reducing patients’ quality of life and causing pain and deficiency in locomotion [6]. HGA has also been found to trigger oxidative stress in AKU [7–10]. Since oxidized lipids are cytotoxic and responsible for initiating inflammatory reactions, a strict correlation between cytotoxicity of the ochronotic pigment and inflammation has be suggested [11]. It has been shown that useful biomarkers for oxidative stress and inflammation in AKU are the Advanced Oxidation Protein Products (AOPP), the products of the oxidation reaction between plasma proteins and oxidizing agents [12–14].

Recent studies have classified AKU as a secondary amyloidosis [11, 15–18], characterised by deposition of serum amyloid A (SAA) fibers, which in its soluble form is a circulating protein produced during chronic inflammatory processes. Studies on AKU patients’ samples (cartilage, salivary glands, chondrocytes and synoviocytes) showed that ochronotic pigment and amyloid fibers share the same location, confirming that SAA is associated with the ochronotic pigment derived from HGA [15]. Under normal conditions SAA is found at low concentrations in plasma (4–6 mg/L), while inflammatory stimulus or tissue damage increase SAA plasma levels 100–1000 times [19], making SAA a sensitive biomarker of inflammation [19]. On top of SAA deposition, SAA plasma level have also been reported to be high in AKU patients ([11, 12, 15–18, 20].

Chitotriosidase (CHIT1) is a chitinase mainly expressed in the differentiated and polarized macrophages [21]. CHIT1 serum concentration correlates with the progression or the severity of several diseases (sarcoidosis, rheumatoid arthritis, ankylosing spondylitis, uveitis, idiopathic pulmonary fibrosis, scleroderma-associated interstitial lung diseases, and chronic obstructive lung diseases), suggesting a potential use of CHIT1 as an AKU biomarker [20, 21].

The major obstacle in carrying out clinical research on AKU is the lack of a standardized methodology to assess disease severity and response to treatment [22], which is complicated by the fact that AKU symptoms differ from an individual to another and no correlation between specific *HGD* mutations and disease severity has been observed so far [5, 23]. A reliable way to monitor patients’ clinical condition and overall health status is the use in clinical practice and research of measures of quality of life (QoL) [20, 24]. QoL allows to observe the evolution of diseases from acute to chronic, and to assess the suitability of the therapeutic interventions considering patients’ symptoms, general health status and care satisfaction [24].

Our previous studies showed that, in a rare and multisystemic disease like AKU, QoL scores help to identify health needs and to evaluate the impact of disease [20, 25], suggesting the presence of a correlation between QoL and the clinical data deposited in the ApreciseKUre database, which could be instrumental in shading light on AKU complexity. Here we have developed a machine learning application that perform a prediction of the QoL scores based on clinical data deposited in the ApreciseKUre. We believe this approach can be turned into a best practice model also for other rare diseases and can be useful for overcoming the obstacles in small dataset management and analysis.

## Materials and methods

### Patient data

The ApreciseKUre contains data from 203 patients, but only 129 have a complete and comprehensive set of information, which have been used in this study [26–28]. ApreciseKUre contains information about biomarkers and replies to questionnaires (for a full description of data deposited in ApreciseKUre see [20]. Patients data are classified according with 11 QoL scores: (i) physical health score, (ii) mental health score, (iii) AKU Severity Score Index (AKUSSI) joint pain, (iv) AKUSSI spinal pain, (v) Knee injury and Osteoarthritis Outcome Score (KOOS) pain, (vi) KOOS symptoms, (vii) KOOS daily living, (viii) KOOS sport, (ix) KOOS QOL, (x) Health Assessment Questionnaire Disability Index (HAQ-DI) and (xi) global pain visual analog scale (hapVAS). (for more details see Additional file 1).

### Statistical analysis and machine learning

- Preliminary statistical analysis

The input data were firstly examined through a preliminary statistical analysis. A correlation matrix based on Pearson correlation coefficient was calculated to measure the linear correlation between QoL scores:

$$ -\mathbf{1}\frac{{\boldsymbol{\upsigma}}_{\mathbf{xy}}}{{\boldsymbol{\upsigma}}_{\boldsymbol{x}}{\boldsymbol{\upsigma}}_{\boldsymbol{y}}}=\frac{\sum_{\boldsymbol{i}=\mathbf{1}}^{\boldsymbol{n}}\left({\boldsymbol{x}}_{\boldsymbol{i}}-{\boldsymbol{\upmu}}_{\boldsymbol{x}}\right)\left({\boldsymbol{y}}_{\boldsymbol{i}}-{\boldsymbol{\upmu}}_{\boldsymbol{y}}\right)}{\sqrt{\sum_{\boldsymbol{i}=\mathbf{1}}^{\boldsymbol{n}}{\left({\boldsymbol{x}}_{\boldsymbol{i}}-{\boldsymbol{\mu}}_{\boldsymbol{x}}\right)}^{\mathbf{2}}}\sqrt{\sum_{\boldsymbol{i}=\mathbf{1}}^{\boldsymbol{n}}{\left({\boldsymbol{y}}_{\boldsymbol{i}}-{\boldsymbol{\mu}}_{\boldsymbol{y}}\right)}^{\mathbf{2}}}}\mathbf{\le}+\mathbf{1} $$

where σ_xy_ is the covariance of the two variables x and y, σ_x_ and σ_y_ are the variances of x and y, respectively, and μ_x_ and μ_y_ are the mean values.

### Application of different ML algorithms

**Fig. 1:**
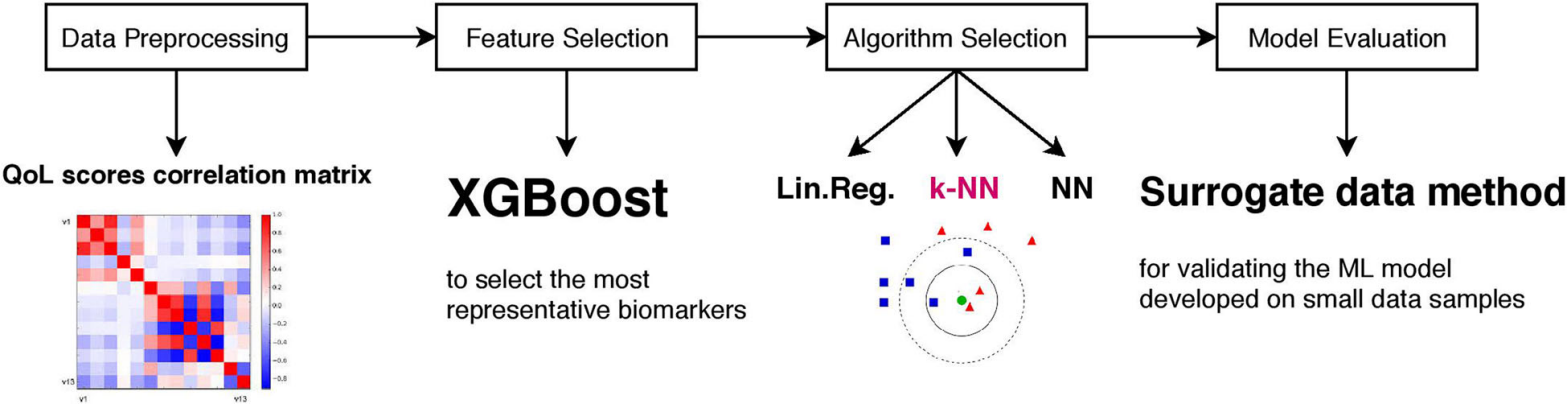
Machine learning framework. A 4-steps workflow of the machine learning-based classification model

Machine learning (ML) is an algorithm-based novel modeling technique that has been introduced recently to select key behavior features (biomarkers) and predict risk levels [29]. ML methods are more precise and accurate in terms of prediction abilities compared with traditional statistical methods, because complex intervariable interactions are taken into account in ML only [30]. There are several key steps of the machine learning-based classification model: data preprocessing, feature selection, algorithm selection and model evaluation. Our workflow is described in Fig. 1.

In this study, to select the most representative predictors (among biomarkers included in ApreciseKUre) for QoL scores we have applied Extreme Gradient Boosting (XGBoost). It is a key algorithm in the processes of clustering evaluation, resampling evaluation, feature selection and prediction, [31] able to calculate variable importance defined as the statistical significance of each variable with respect to its effect on the generated model [32]. Starting from selected biomarkers, QoL score prediction is then evaluated comparing the performance of three other different ML techniques: (i) Linear Regression [33], (ii) Neural networks [34], and (iii) K-nearest neighbours algorithm (k-NN) [35].). Finally, we applied a surrogate data method [36].

## Results

### QoL scores statistical correlation

**Fig. 2:**
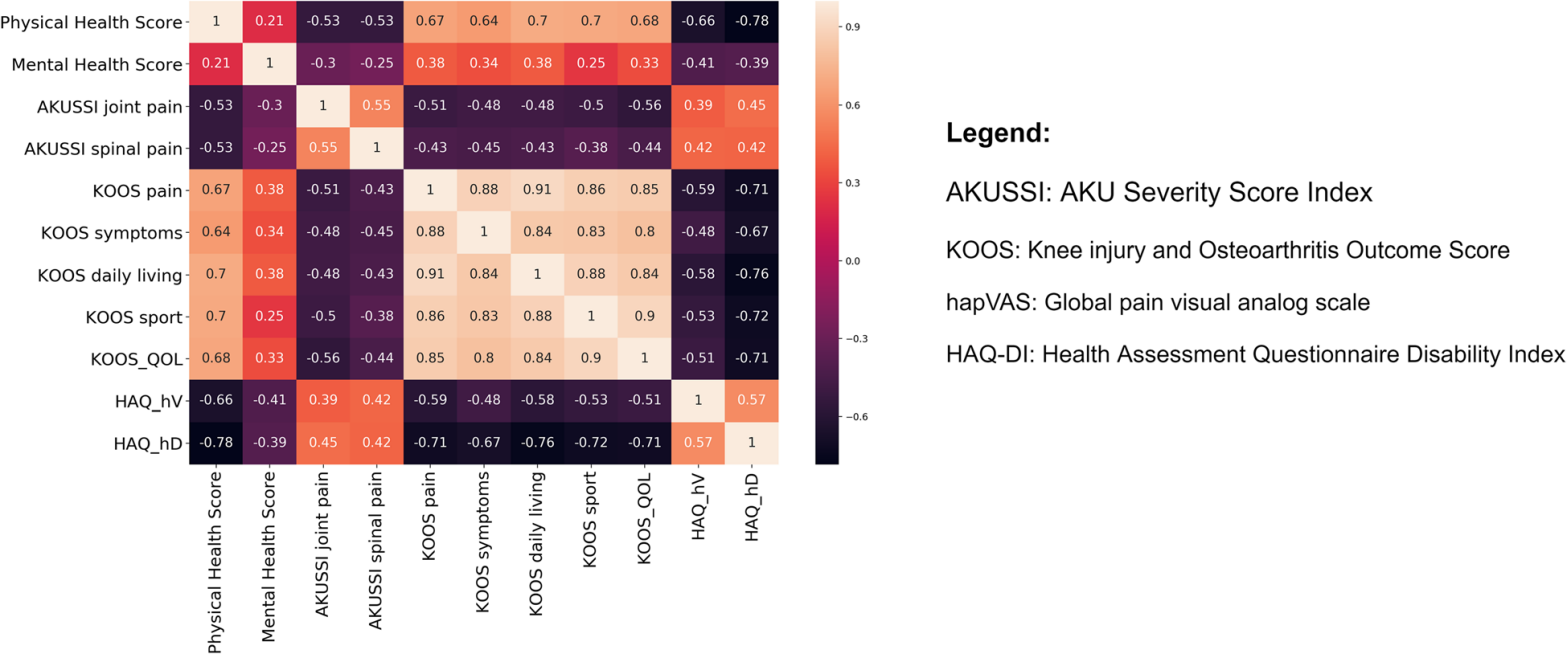
Correlation matrix of health survey questionnaires. In this correlation matrix all QoL scores are correlated to each other. In black statistically significant inverse correlation, in light-pink statistically significant direct correlation, in red or purple not statistically significant correlations

In the present study, a machine learning algorithm was implemented with the aim to perform a prediction of QoL scores based on 129 patients’ clinical data deposited in the ApreciseKUre database [26, 27]. QoL scores were firstly examined through a preliminary statistical analysis in order to evaluate the degree of correlation among pairs of variables (Fig. 2).

It is interesting to notice the presence of correlation among AKUSSI, KOOS and HAQ scores. Specifically, KOOS pain, KOOS symptoms, KOOS daily living and KOOS sport have a high correlation with AKUSSI joint pain and spinal pain, and with hapVAS and HAQ-DI. Differently, the mental health score correlation with all the other QoL scores is not statistically significant (between − 0.3 and 0.3). Taken together, these data suggest that the mental health score, the only one assessing the psychological status of the patient, is independent from other QoL scores linked to the individual’s physical status. Surprisingly, this finding shows that the patients’ psychological experience, based on the evaluation of levels of anxiety and depression, is not directly related with their actual physical and clinical status.

### AKU biomarkers selection using XGBoost

**Fig. 3:**
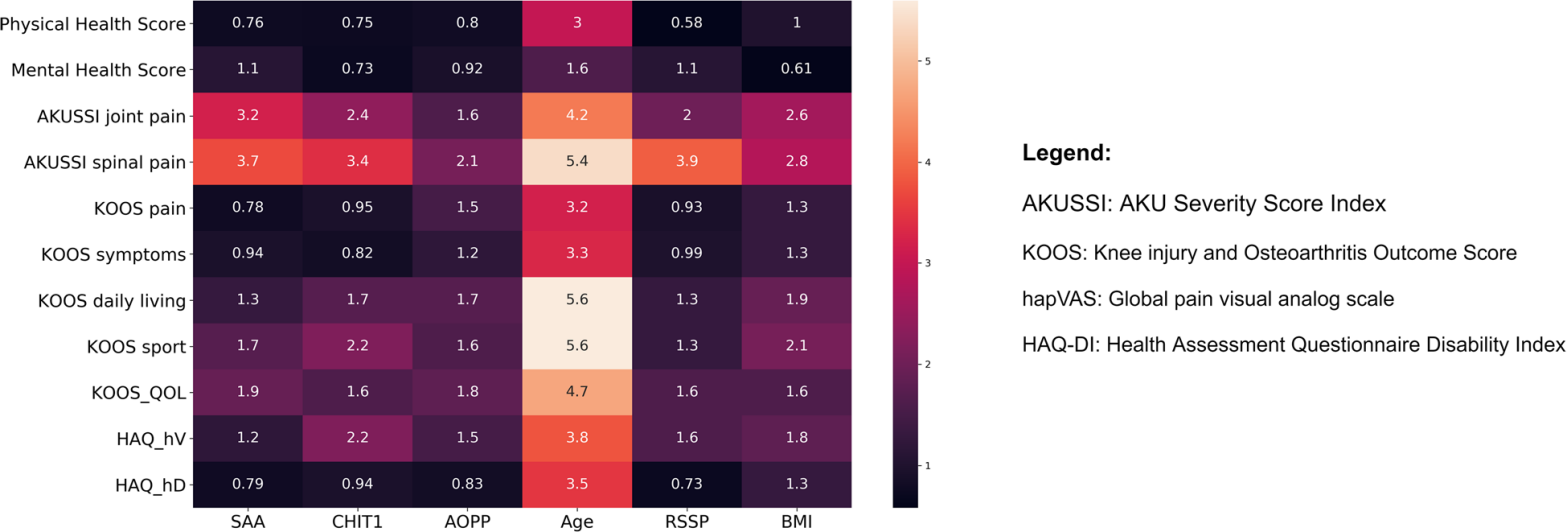
Variable importance Xgboost for each QoL score. In the matrix are reported all the most representative indicators (X axes) with respect to Qol scores (Y axes) for scores prediction with their corresponding variable importance. Color scale goes from the lower value (in black) to highest value (light pink)

Selection of the most representative predictors for QoL scores was performed by Extreme Gradient Boosting. XGBoost reveals that the most statistically significant variables among 110 biomarkers included in ApreciseKUre [27] are: age, SAA, CHIT1, AOPP, RSSP, BMI. Variable importance scores of the above mentioned six best biomarkers, with respect to every QoL score, are reported in Fig. 3.

### ML algorithm selection

Based on these preliminary analyses, different ML models (Linear Regression, Neural networks and k-NN) were implemented to improve the correlation analysis of biomarkers and QoL score. The ML models were compared based on RAE (Relative Absolute Error) indicator (Table 1) and R^2^ score (Coefficient of determination):

**Table 1:** ML algorithm performance comparison

Model	RAE	R^2^
Linear Regression	0.34	0.87
Neural networks	0.28	0.91
k-NN	0.25	0.94

Comparison based on RAE and R^2^ score among different ML models. K-NN resulted to have the lowest RAE, thus the best performance

**Fig. 4:**
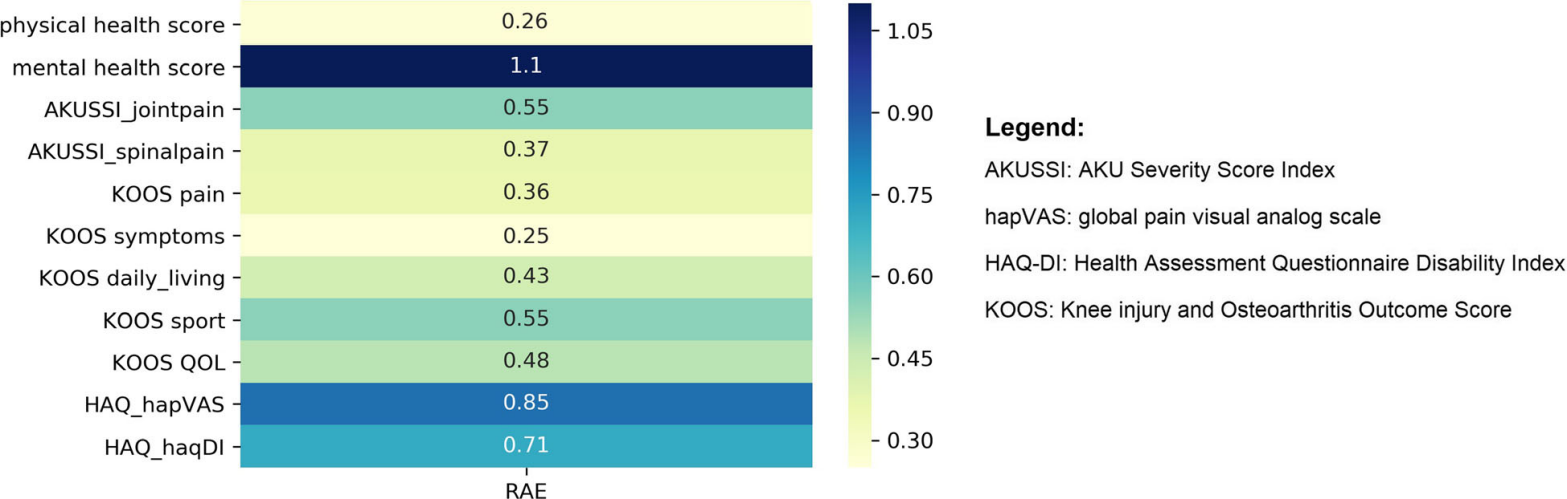
Performance for each QoL Score. Representation of model accuracy (RAE) for each QoL score, scale from the lower value (in light green) to highest value (blue)

As such, k-NN resulted to be the most accurate algorithm to predict QoL scores. Therefore, we performed a k-NN on each of the 11 QoL scores and KOOS symptoms score showed the most accurate prediction (lowest RAE: 0.25) (Fig. 4). Conversely, mental health scores might not be predicted with a sufficient accuracy (highest RAE: 1.1), indicating limited or no connection with age, SAA, CHIT1, AOPP, RSSP, BMI values, which is in line with our preliminary statistical analysis.

Differently from other scores (AKUSSI, KOSS, HAQ, hapVAS), mental health score is measured across eight domains (vitality, physical functioning, bodily pain, general health perception, physical role functioning, social functioning, emotional role functioning, mental health), thus it is not unexpected that there is not a correlation with age and other AKU biomarkers. This observation, in line with [20], confirms a not infrequent disability paradox in inherited/chronic disease, underlying the difference between the physical and mental impact on disease severity, which may underestimate overall mental state.

The obtained results demonstrated the power of ML techniques in extrapolating information from a biomarkers dataset to make predictions of QoL scores. ML, with their remarkable ability to derive meaning from complicated or imprecise data, can be used to extract patterns and detect trends that are too complex to be noticed by either humans or other computer techniques. For instance, in Fig. 3, age, SAA, CHIT1, AOPP, RSSP, BMI related to AKUSSI spinal pain and AKUSSI joint pain scores assumed the highest variable importance, suggesting the hypothesis they would have been the best QoL indicators. However, as shown in Fig. 4, AKUSSI spinal score and AKUSSI joint pain RAE for k-NN prediction resulted to be higher in comparison with KOOS symptom. Additionally, HAQ hapVAS and HAQ-DI showed high RAE despite the biomarkers variable importance is not different from KOOS symptom score. In view of this, based on the k-NN prediction, KOOS symptoms can be considered as a useful guide for better understanding symptoms and difficulties experienced by patients.

In conclusion, a k-NN based on the combination of parameters like age, SAA, CHIT1, AOPP, RSSP and BMI was able to predict with low RAE the value of KOOS symptoms. Taken singularly these features are not predictive and it is already well known that parameters like age, SAA, CHIT1 are linked with disease severity. The innovative finding of the present work is that, for the first time, we have found an ensemble of multiple complementary features (SAA, CHIT1, AOPP, RSSP, related with inflammation, oxidative stress, amyloidosis; age and BMI, linked with lifestyle) whose combination produce better k-NN prediction results than any single one.

### Validating ML models using surrogate data

Small dataset conditions and the associated random effects make validation of ML models a challenging task. For these reasons, to validate the obtained model, we applied a surrogate data method, which has been previously shown to be the most suitable method for small dataset [36]. In this approach, the surrogate data were generated from random numbers able to mimic the distribution of the original dataset independently for each component of the input. They statistically resemble the original data in terms of their mean, standard deviation and range, but they do not maintain the complex relationships between the variables of the real dataset (Table 2).

**Table 2:** Correlation matrix of original and surrogate dataset

ORIGINAL	Pearson correlation coefficient						
	Variables	SAA	CHIT1	AOPP	RSSP	age	BMI
	SAA	1.00	−0.01	− 0.01	0.15	0.02	0.23
	CHIT1	−0.01	1.00	0.00	0.28	0.40*	−0.01
	AOPP	−0.01	0.00	1.00	0.06	0.09	0.17
	RSSP	0.15	0.28	0.06	1.00	0.38*	0.09
	Age	0.02	0.40*	0.09	0.38*	1.00	0.14
	BMI	0.23	−0.01	0.17	0.09	0.14	1.00
	*p*-value						
	Variables	SAA	CHIT1	AOPP	RSSP	age	BMI
	SAA	0.00	0.56	1.00	0.11	0.57	0.01
	CHIT1	0.56	0.00	0.87	0.00	0.00	0.86
	AOPP	1.00	0.87	0.00	0.69	0.45	0.10
	RSSP	0.11	0.00	0.69	0.00	0.00	0.59
	Age	0.57	0.00	0.45	0.00	0.00	0.28
	BMI	0.01	0.86	0.10	0.59	0.28	0.00
SURROGATE	Pearson correlation coefficient						
	Variables	SAA	CHIT1	AOPP	RSSP	age	BMI
	SAA	1.00	−0.16	0.02	0.22	−0.02	−0.16
	CHIT1	−0.16	1.00	−0.03	−0.06	−0.08	0.06
	AOPP	0.02	−0.03	1.00	−0.12	0.06	−0.01
	RSSP	0.22	−0.06	−0.12	1.00	−0.18	0.09
	Age	−0.02	−0.08	0.06	−0.18	1.00	−0.10
	BMI	−0.16	0.06	−0.01	0.09	−0.10	1.00
	*p*-value						
	Variables	SAA	CHIT1	AOPP	RSSP	age	BMI
	SAA	0.00	0.72	1.00	0.23	0.57	0.10
	CHIT1	0.72	0.00	0.88	0.02	0.00	1.00
	AOPP	1.00	0.88	0.00	0.66	0.61	0.20
	RSSP	0.23	0.02	0.66	0.00	0.02	0.58
	Age	0.57	0.00	0.61	0.02	0.00	0.28
	BMI	0.10	1.00	0.20	0.58	0.28	0.00

The first table shows the Pearson correlations coefficients and the *p*-values of our original dataset, the second table shows the Pearson correlations coefficients and the *p*-values of surrogate dataset

*indicates statistically significant values

Therefore, real-data models are expected to perform significantly better than the surrogate data models [36]. The same k-NN algorithm was applied to both datasets, which were randomly split into 80–20% for, respectively, the training and test sets. Each model was trained and validated on 1000 different runs, each using a different training sets, selecting a 10% of the training set to validate the model. The performances of the model, in terms of RAE and R^2^ score, were calculated as the average over the runs.

**Fig. 5:**
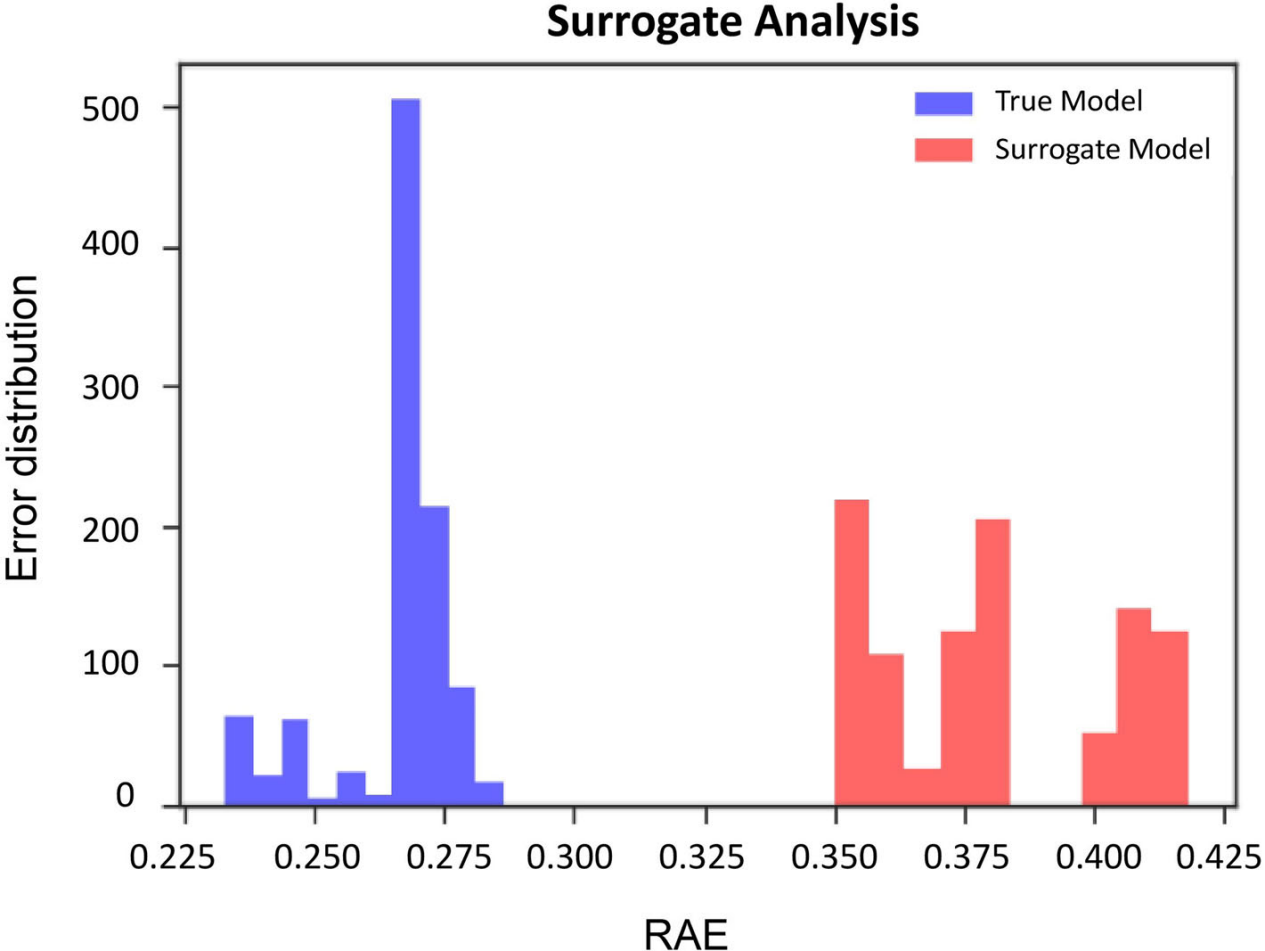
Surrogate Test Analysis. Comparison of performance based on RAE values, between k-NNs trained on surrogate data (red) and original dataset (blue)

The models trained on our real biochemical and clinical dataset achieve an increase in the average of predictive performance than analogous models trained on the surrogate controls. Indeed, the error distribution of surrogate-model (RAE 0.38) was unequivocally higher than the true-model one (RAE of 0.25) confirming the consistency of our dataset. Thus, it is possible to conclude that the obtained predictive method is not biased or resulting from an overfitting of the model on a small-sized dataset (Fig. 5). This framework allowed ML algorithms to successfully predict clinical and QoL scores outcomes despite small datasets.

## Discussion

The limited number of AKU patients spread around the world represent a major obstacle for generating a standardized strategy to assess disease stage and progression. While several biomarkers for AKU have been identified, a clear connection between biomarkers levels and disease severity (QoL score) is still missing. Here, we implemented an ML method from which QoL of AKU patients can be predicted based on age, oxidative stress (AOPP and RSSP), amyloidosis (SAA) inflammation (CHIT1) biomarkers and BMI, while HGA appears to be extremely variable and unrelated with disease severity. An intricate and complex pattern of oxidative stress, amyloidosis and inflammation is evidently the main important indicator of patients’ health status.

Moreover, QoL scores worsen progressively with the age. Aging is associated to decrease antioxidant defenses (for instance the age-related decline in plasma glutathione (GSH) and low molecular weight thiols) and increase ROS production, allowing oxidatively damaged macromolecules to accumulate [37]. AKU subjects undergo a significant decrease in serum free protein thiols and a significant increase in low molecular weight mixed-protein thiols with aging [38].

Our ML model suggested that KOOS indicators could be used to better understanding symptoms and difficulties experienced by AKU patients.

KOOS is a valid, reliable and responsive tool to evaluate both short-term and long-term consequences of knee injury and primary OA. It is a patient-reported outcome measurement, developed to assess the opinion of patients about their knees and associated problems, and it is routinely used for follow-up evaluations [39]. Multiple studies in patients with knee injury and knee OA report that the KOOS demonstrates expected convergent and divergent construct validity, with the KOOS more strongly correlated with subscales of the ShortForm- 36 (SF-36) that measure similar constructs [40]. This is the reason why KOOS prediction could be potentially useful to assess consequences of primary OA, to evaluate changes from week to week induced by treatment (medication, surgery, physical therapy) or over the years due to a primary knee injury, posttraumatic OA or primary OA [39], to identify the main important prognostic biomarkers of AKU, to help the clarification of physiopathological mechanisms of AKU and ochronosis, and to assess the efficacy of future pharmacological treatments.

Similarly, AOPP and RSSP, indicators of oxidative stress and inflammation, have shown to influence the k-NN model. This is not surprising since AKU patients undergo a significant increase in RSSP with aging [38]. Such a trend suggests that progression of AKU symptoms could be related to impaired anti-oxidant status [10]. HGA induces a significant oxidation of a number of serum and chondrocyte proteins. Further investigations allowed highlighting how HGA-induced proteome alteration, lipid peroxidation, thiol depletion, and amyloid production could contribute to oxidative stress generation and protein oxidation in AKU [7]. Furthermore, this is in line with our findings that SAA can be considered as an AKU biomarker for amyloidosis [15]. In fact, a chronic inflammatory status paralleled by inadequate antioxidant defenses is known to promote the aberrant production of amyloidogenic proteins, ultimately leading to secondary amyloid deposition [7]. SAA-amyloidosis colocalizes with ochronotic pigment as well as with tissue calcification, lipid oxidation, macrophages infiltration, cell death, and tissue degeneration [11, 16, 17].

One of the most striking results is that, differentially from the physical QoL scores based on bodily pain scales and general factor of physical health, mental health status is not predictable by k-NN using the biomarkers listed above. It is measured across eight domains: vitality, physical functioning, bodily pain, general health perception, physical role functioning, social functioning, emotional role functioning, mental health. Surprisingly, in line with the study of [20], the level of biomarkers reported to be directly linked to physical status and pain are not influencing social functioning, role-emotional, levels of depression and anxiety [20]. In conclusion, the outcome of our work was that, for the first time, we have found a biomarkers combination which, in accordance with literature, was able to produce reliable k-NN prediction results. Thanks to this ML algorithm, we will be able to correctly predict KOOS symptoms of a new AKU patient just relying on clinical and lifestyle data.

### Current study limitations and future perspective

There are several challenges in studying an ultra-rare and complex disease like AKU, and specifically (i) the paucity of specimens and available data, and (ii) the lack of a standardized method able to objectively assess disease severity or response to treatment. For this reason we developed ApreciseKUre database, aiming to collect as many AKU patients’ data as possible, and to use QoL scores to monitor patients’ clinical condition and health status, although the database does not yet include objective disease severity findings (i.e. imaging, cardiac valve or calcification, radiographic severity score, treatment modalities, time to surgery, etc). We believe that this study could be a starting point for a better investigation of the utility and reliability of QoL scores, which are becoming increasingly popular, and their correlation to biochemical and clinical biomarkers. For example, the AKUSSI score, which incorporates into a single score multiple clinically meaningful AKU outcomes, medical photography imaging investigations and detailed questionnaires, performed poorly in the model based on the selected biomarkers (AKUSSI joint pain RAE 0.37 and AKUSSI spinal pain RAE 0.55). However, as shown in Fig. 3, parameters like age, SAA, CHIT1, AOPP, RSSP, BMI were the 6 variables with the highest importance values. In literature, these 6 variables have been already used as biomarkers for AKU. In fact, there is an intimate connection between HGA and the ochronotic process, SAA and amyloidosis, inflammation and oxidative stress in AKU, as demonstrated by structural co-localization of ochronotic pigment and SAA-amyloid and co-localization of SAA with crucial cytoskeletal proteins in AKU chondrocytes [20]. As described in [12], some AKU patients, who underwent joint replacement surgery and complained about articular disorders, arthropathy and joint pain together with other co-morbidities, showed pathological levels of SAA and AOPP above the reference value. Moreover, serum concentration of SAA [41, 42] and CHIT1 activity [43, 44] are markers of disease severity in several rheumatic conditions, and in [20] was provided the evidence that AKU patients present significantly high SAA and chitotriosidase activity in comparison with controls. Some objective disease severity findings, such as cardiac valve calcification and treatment modalities, are strictly linked with amyloidosis, inflammation and oxidative stress. For example, in [11, 16, 17], SAA deposition was detected by immunofluorescence technique in AKU aortic valve and it was tested that low dose methotrexate can down-regulate inflammation and lower SAA production in AKU [20].

In a complex disease like AKU, also lifestyle parameters like BMI are not neglectable. As shown in Table 2, SAA and AOPP have a weak direct correlation with BMI (*p*-value respectively 0.01 and 0.10), which in turn increases with age. It has been previously shown that oxidative stress increase with a rising BMI, as a consequence of an impaired antioxidant status [20, 45] through various biochemical mechanisms, such as superoxide generation from NADPH oxidases, oxidative phosphorylation and glyceraldehyde auto-oxidation [46]. Moreover, in line with [20], a positive association was found between SAA and BMI, since in obesity (where low-grade inflammation is found) adipose tissue is the major source of SAA, which can be considered an obesity-related inflammatory protein [47, 48].

Age is an important driving factor for the prediction of QoL scores and it is a common observation that clinical symptoms might worse with aging. In fact, as shown in Table 2, CHIT1 and RSSP correlate with age (p-value 0.0 for both biomarkers). This is confirmed by the fact that when age is removed from the set of six biomarkers (SAA, CHIT1, AOPP, RSSP, BMI) able to predict QoL scores, the k-NN RAE of KOOS symptoms jump to 0.31. Unfortunately, it is not easy to gather data of very young patients, since people start showing AKU symptoms in their 30/40s, even if the dark discoloration of the urine is present from birth. The systematic use of the ApreciseKUre database will increase the number of patients and will allow us to develop an upgraded version of our algorithm to include an adjustment for the age of the patients.

It is important to specify that this study was based on baseline biochemical and clinical analysis, since the very limited number of information regarding the longitudinal changes, changes during the acute phase, medication effects, differences after joint replacement did not produce robust statistical results. Being AKU a chronic but not lethal disease, the future direction of our study will aim at collecting more AKU follow-up patients’ data before and after treatments, in order to evaluate the effectiveness of different therapies. This will be an essential point for a typical precision medicine approach, in which each patient is closely monitored over time and several types of information are collected to understand the uniqueness of each individual. This predictive system will allow for the easy monitoring of AKU disease evolution and it will help clinicians in the selection of the most appropriate treatment, and evaluate its efficacy by observing the trend of QoL scores and biomarkers. In summary, this cost-effective computational method will be beneficial in supporting experimental and clinical studies and, at the same time, will help patients by identifying the most promising treatments.

## Conclusion

In conclusion, the combination of a ML to analyse and re-interpret data available in the ApreciseKUre shows the potential direct benefits for patient care and treatments, highlighting the necessity of patient databases for rare diseases, like ApreciseKUre. We believe this is not limited to the study of AKU, but it represents a proof of principle study that could be applied to other rare diseases, allowing data management, analysis and interpretation.

## Supplementary information

**Additional file 1.** In Additional file 1 a more detailed description of QoL scores is provided. Moreover, informational layers, data and features included in ApreciseKUre are collected and listed.

## Data Availability

The datasets generated and/or analysed during the current study are available in the ApreciseKUre repository, [http://www.bio.unisi.it/aku-db/]. The datasets used and/or analysed during the current study are available from the corresponding author on reasonable request.

## Abbreviations

aimAKU: Italian Association of Alkaptonuric patients
AKU: Alkaptonuria
AKUSSI: AKU Severity Score Index
AOPP: Advanced Oxidation Protein Products
BMI: Body Mass Index
CHIT1: Chitotriosidase
GSH: glutathione
hapVAS: global pain visual analog scale
HAQ-DI: Health Assessment Questionnaire Disability Index
HGA: homogentisic acid
HGD: Homogentisate 1,2-dioxygenase
IL-1: Interleukin-1
IL-6: Interleukin-6
k-NN: K-nearest neighbors algorithm
KOOS: Knee injury and Osteoarthritis Outcome Score
ML: Machine learning
OA: Osteoarthritis
QoL: Quality of life
RAE: Relative Absolute Error
RSSP: S-thiolated proteins
SAA: Serum amyloid A
SF-36: Short Form-36 questionnaire
SOFIA: Subclinical Ochronotic Features In Alkaptonuria
SONIA1: Suitability Of Nitisinone In Alkaptonuria 1
SONIA2: Suitability of Nitisinone in Alkaptonuria 2
TNF: Tumor necrosis factor
XGBoost: Extreme Gradient Boosting
