# Supplementary material for: Machine learning application for development of a data-driven predictive model able to investigate quality of life scores in a rare disease
Source: Orphanet J Rare Dis. 2020 Feb 12;15:46. doi: 10.1186/s13023-020-1305-0 (PMC7017449; doi:10.1186/s13023-020-1305-0)
Supplement: Supplementary file 1 — Additional file 1. In Additional file 1 a more detailed description of QoL scores is provided. Moreover, informational layers, data and features included in ApreciseKUre are collected and listed. [file 13023_2020_1305_MOESM1_ESM.docx]

**Supplementary Materials**

The data used in this study included:

*1) Health Survey Questionnaires:*

- *The Short Form-36 (SF-36) questionnaire:* is a multi-purpose short-form health survey with 36 questions that measures patients’ QoL across several domains, which are both physically- and emotionally- based (Braconi, et al., 2018). Both physical and mental status scores measure patients' quality of life across eight domains: vitality, physical functioning, bodily pain, general health perception, physical role functioning, social functioning, emotional role functioning, mental health. A score of “0” indicates maximum disability, while a score of “100” indicates no disability (Braconi, et al., 2018) The survey does not take into consideration a sleep variable and has a low response rate in the >65 population (Andresen et al. 1999).
- *The Knee injury and Osteoarthritis Outcome Score (KOOS).* The KOOS is a knee-specific instrument developed with the purpose of evaluating short- and long-term symptoms and function in subjects with knee injury and osteoarthritis and of assessing associated problems. (Roos & Lohmander, 2003) (Braconi, et al., 2018).
- *The HAQ-DI questionnaire:* it includes a disability index (HAQ-DI) and a and global pain visual analog scale (hapVAS) (Bruce & Fries, 2003) (Braconi, et al., 2018).
- *AKU Severity Score Index (AKUSSI):* questionnaire-based evaluation of AKU severity (Ranganath & Cox, 2011). It incorporates multiple, clinically meaningful AKU outcomes combined with imaging investigations, medical photography and detailed questionnaires into a single score with the objective of understanding and describing the features of AKU (Braconi, et al., 2018).

*2) Biomarkers*

- serum amyloid A (SAA) is a biomarker for inflammation (Gabay & Kushner, 1999) (Braconi, et al., 2018);
- *Chitotriosidase (CHIT1*) is a biomarker for chronic inflammation (Cho, Weiden, & Lee, 2015);
- Advanced Oxidation Protein Products (AOPP) is a biomarker for oxidation stress and level of inflammation, which, as mentioned before, in AKU are intimately linked (Braconi, et al., 2016);
- *S-thiolated proteins (RSSP)* is a biomarker for oxidation stress (Giustarini, 2011).
- *Body Mass Index (BMI)* is an indicator of patient lifestyle.

ApreciseKUre (available at <http://www.bio.unisi.it/> after user registration) is a database framework developed and implemented for the collection of information from patients with AKU. This represents a unique integrated and interactive online tool that includes an automatic system of data analysis. In the online platform it is in fact possible to compute correlations of the patients features (based on the Pearson correlation coefficient) and other statistical analysis, and this allows for the investigation and integration of different types of data together in a unique framework. The available data are divided in several information layers (Demographics and Genetics, Oxidative stress, inflammation and amyloidosis biomarkers, Lifestyle, Quality of Life Scores, Concomitant Diseases, Drugs, Blood Analysis, Urine Analysis, Plasma Analysis, Histopathology) included in a unique environment that makes it suitable for the analysis of the disease. The database is composed of a large cohort of AKU patients for a total amount of 203 individuals:

- 25 patients from Associazione Italiana Malati di Alkaptonuria;
- 39 patients derived from Suitability of Nitisinone In AKU (SONIA) 1, forming one of the 3 steps of the clinical trial called DevelopAKUre;
- 139 derived from SONIA 2, the second step of DevelopAKUre.

The presence of this large cohort could have important applications for the studies of this ultrarare disease and other common rheumatic disorders, particularly toward a Precision Medicine approach. Each patient in the ApreciseKUre database is characterized by more than 100 features, describing clinical, genotypic and metabolic information.

Currently, this study was based on baseline biochemical and clinical analysis since the number of information regarding the longitudinal changes, changes during the acute phase data, medication effects, differences after joint replacement are not enough to provide a robust statistical result.

The informational layers and the features are listed in the following tables:

| Demographics and Genetics |
| --- |
| id_patient |
| id_in_hgd_gene_mutation_database |
| Gender |
| date_of_birth |
| protein_change_allele_1 |
| dna_change_allele_1 |
| exon/intron_allele_1 |
| protein_change_allele_2 |
| dna_change_allele_2 |
| exon/intron_allele_2 |
| secondary_structure_allele_1 |
| specific_activity_allele_1 |
| specific_activity_allele_2 |
| Country |
| Relatives |
| father's_origin |
| mother's_origin |
| who_is_affected_in_family |
| year_diagnosis_AKU |

| Oxidative stress, inflammation and amyloidosis biomarkers | |
| --- | --- |
| saa | Serum Amyloid A microg/mL (ELISA) |
| chitotriosidase | chitotriosidase nmol/h/ml (ELISA) |
| serum_hga | Homogentisic acid microM (ELISA) |
| hga_hplc | Homogentisic acid mmol/L (HPLC) |
| catd | Cathepsin D ng/mL (ELISA) |
| il-6 | Interleukin-6 pg/mL (ELISA) |
| il-1beta | Interleukin-1 Beta pg/mL (ELISA) |
| il-1ra | Interleukin Receptor antagonist pg/mL (ELISA) |
| tnf_a | Tumor necrosis factor alfa pg/mL (ELISA) |
| crp | C-reactive protein mg/dL) |
| crp_elisa | C-reactive protein microg/mL (ELISA) |
| mmp3 | Matrix metalloproteinase 3 ng/mL (ELISA) |
| aopp | Advanced oxidation protein products micromol/dL (ELISA) |
| PSH | Serum protein thiols micromol |
| CySSP | Protein/cysteine mixed disulphides micromole |
| CyGlySSP | Protein/cysteineglycine mixed disulphides micromol |
| HcySSP | Protein/Homocysteine mixed disulphides micromol |
| yGluCySSP | Protein/Gamma-Glutamylcysteine mixed disulphides micromol |
| GSSP | Protein/Glutathione mixed disulphides micromol |
| RSSP | S-thiolated proteins micromol |
| PTI | Protein Thiolation Index |

| Lifestyle | |
| --- | --- |
| smoker/cigarettes_a_day | N/day |
| alcohol_units_weekly | N/week |
| BMI | Body Mass Index |

| Quality of Life Scores | |
| --- | --- |
| physical_health_score | Physical Health Score |
| mental_health_score | Mental Health Score |
| AKUSSI_jointpain | AKU Severity Score Index joint pain |
| AKUSSI_spinalpain | AKU Severity Score Index spinal pain |
| KOOSpain | Knee injury and Osteoarthritis Outcome Score pain |
| KOOSsymptoms | Knee injury and Osteoarthritis Outcome Score symptoms |
| KOOSdaily_living | Knee injury and Osteoarthritis Outcome Score daily living |
| KOOSsport | Knee injury and Osteoarthritis Outcome Score sport |
| KOOS_QOL | Knee injury and Osteoarthritis Outcome Score Quality of Life |
| hapVAS | Global pain visual analog scale |
| HAQ-DI | Health Assessment Questionnaire Disability Index |

| Concomitant Diseases | |
| --- | --- |
| arterial_hypertension | Y/N |
| hyperlipoproteinemia | Y/N |
| hypothyroidism | Y/N |
| asthma | Y/N |
| diabetes_mellitus | Y/N |
| hypercholesterolemia | Y/N |
| ochronotic_arthropathy | Y/N |
| osteoporosis | Y/N |
| other_diseases | Y/N |

| Drugs | |
| --- | --- |
| anti_inflammatories | Y/N |
| ATC_antinflammatories | Anatomical Therapeutic Chemical Classification Code |
| painkillers | Y/N |
| ATC_painkillers | Anatomical Therapeutic Chemical Classification Code |
| other_drugs | Y/N |
| ATC_code | Anatomical Therapeutic Chemical Classification Code |

| Blood analysis | |
| --- | --- |
| glucose | mg/dL |
| creatinine | mg/dL |
| cholesterol | mg/dL |
| triglycerides | mg/dL |
| hdl_cholesterol | mg/dL |
| ldl_cholesterol | mg/dL |
| alkaline_phosphatase | UI/L |
| cystatin_C | mg/dL |

| Urine Analysis | |
| --- | --- |
| urines_code | Code |
| date_urines | Date |
| creatinine_urines | mg/dl |
| hga_urines | Homogentisic acid mg/24h |
| bqa | Benzoquinone acetic acid mg/dl |
| urate | Urate mg/dl |
| uric_acid_urine | Urine Acid mg/dl |
| tyr_urines | Tyrosine mg/dl |
| hypoxanthine_urines | Hypoxanthine urines mg/dl |
| xanthine_urines | Xanthine urines mg/dl |

| Plasma Analysis | |
| --- | --- |
| id_plasma | Code |
| date_plasma | Date |
| creatinine_plasma | Creatinine in plasma micromol/L |
| hga_plasma | Homogentisic acid micromol/L |
| tyr | Tyrosine micromol/L |
| phe | Phenylalanine micromol/L |
| trp | Tryptophan micromol/L |
| hypoxanthine | Hypoxanthine micromol/L |
| xanthine | Xanthine micromol/L |
| uridine | Uridine micromol/L |
| uric_acid | micromol/L |

| Histopathology | |
| --- | --- |
| congo_red | slide image |
| alizarin_red | slide image |
| oarsi_grade | slide image |
